# La *pandemia diabete* in Italia

**DOI:** 10.1007/s40619-022-01130-4

**Published:** 2022-07-27

**Authors:** Enzo Bonora

**Affiliations:** Endocrinologia, Diabetologia e Malattie del Metabolismo, Universitàà e Azienda Ospedaliera Universitaria Integrata di Verona, Verona, Italia

**Keywords:** Diabete Mellito, Prevalenza, Incidenza, Danno d’organo, Qualità della cura, Organizzazione dell’assistenza

## Abstract

In Italia vivono circa 4 milioni di persone con il diabete e ogni anno ci sono circa 350 mila nuove diagnosi. Le persone con diabete ricevono prescrizioni di farmaci, esami di laboratorio e strumentali e richiedono ricoveri ospedalieri più spesso delle persone senza il diabete. Il diabete è gravato da aumentata mortalità e accorcia la vita, soprattutto nelle persone di media età. La qualità della cura nelle persone con diabete è subottimale, soprattutto fra chi non è assistito nei centri diabetologici (che, peraltro, rappresentano dei luoghi “salvavita”). Il diabete contribuisce fortemente alla spesa sanitaria e comporta ingenti spese, non tutte specificamente sanitarie. Nonostante tutto questo e nonostante leggi nazionali e regionali, documenti di indirizzo nazionali e regionali, PDTA regionali e locali, promesse e proponimenti di varia provenienza, la pandemia diabete non riceve le attenzioni che merita.

**Informazioni Supplementari:**

La versione online contiene materiale supplementare disponibile su 10.1007/s40619-022-01130-4.

## Premessa

Negli ultimi 20 anni, in più circostanze è stato utilizzato il termine pandemia per sottolineare che il diabete stava dilagando in tutto il mondo [1, 2]. Il diabete tipo 2, in particolare, è stato indicato dalla Organizzazione Mondiale della Sanità come la malattia non trasmissibile che, insieme a due malattie trasmissibili (malaria e tubercolosi), era diventata un’emergenza planetaria [3] per una numerosità di casi che aumentava in maniera esponenziale [4] e per costi che stavano diventando elevatissimi [5]. Il diabete, seppure sempre trascurato rispetto ad altre patologie, stava faticosamente guadagnando attenzioni nella seconda decade di questo secolo quando, enorme nei suoi numeri e nella sua drammaticità clinica, sociale ed economica, è arrivata, inattesa e devastante, la pandemia causata dal virus SARS-CoV-2 [6].

Il COVID, tuttavia, per quanto foriero di effetti più severi nelle persone con diabete anche nel nostro Paese [7], non ha però fatto scomparire la *pandemia diabete* che merita a pieno titolo di restare al centro dell’attenzione e di non tornare in una trascuratezza che non è assolutamente meritata da coloro che soffrono per la malattia e da coloro che si adoperano per curarla. In questo articolo sono presentati numeri e concetti che giustificano l’appello a ricordare che mentre la pandemia COVID, prima o poi, fortunatamente, sarà ridotta ai minimi termini, la pandemia diabete purtroppo resterà, interessando circa il 10% della popolazione mondiale.

Nell’articolo si farà riferimento soprattutto a dati provenienti dall’Osservatorio ARNO Diabete, nato circa 15 anni fa e alimentato in seguito dalla collaborazione fra CINECA e Società Italiana di Diabetologia. L’Osservatorio ha prodotto multipli rapporti [8–13] che, basandosi su dati amministrativi (farmaceutica, specialistica ambulatoriale, ricoveri ospedalieri, esenzioni per patologia), hanno mostrato il profilo assistenziale delle persone con diabete che vivono in Italia. Considerando l’elevato numero di persone oggetto dei rapporti (molti milioni) e la provenienza dei dati da ASL appartenenti a regioni del nord, del centro e del sud di Italia, l’Osservatorio fornisce informazioni di notevole affidabilità nella descrizione della situazione nell’intero Paese.

## Prevalenza

Le ultime analisi condotte sull’Osservatorio ARNO Diabete hanno prodotto dati pubblicati nel 2019 e sono basate su dati raccolti nel 2018 su circa 11 milioni di cittadini [13]. Fra questi, circa 700 mila sono risultati essere affetti da diabete, identificato dalle prescrizioni di farmaci specifici (anti-iperglicemici orali e iniettabili), dalla presenza di esenzione per patologia e dalla menzione del diabete fra le diagnosi presenti nella schede di dimissione ospedaliera (SDO). Questi numeri permettono di collocare la prevalenza della malattia diagnosticata al 6,2% [13, 14], un dato simile a quello osservato nel 2014 [11] e nel 2016 [12]. Sembra quindi che, dopo l’aumento importante nella prevalenza osservato nel 2014 rispetto a quanto osservato con dati epidemiologici raccolti con metodica simile nel 1986 [15] e nel 2003 [16], si sia raggiunto una sorta di stato stazionario. Questa prevalenza, estrapolata a circa 61 milioni di residenti in Italia, stimando in circa 0,5% i soggetti con diabete noto ma non rilevabile con i flussi amministrativi in quanto trattati con sola dieta, privi di esenzione per patologia e mai ricoverati con indicazione del diabete fra le diagnosi riportate nelle SDO, permette di stabilire con buona approssimazione che il numero totale dei residenti in Italia con diabete noto ammonti a circa 4 milioni. La ricerca dei casi di diabete non diagnosticato mediante la misurazione della glicemia a digiuno o dopo carico orale di glucosio o della HbA1c aumenterebbe il numero dei casi. Studi condotti negli anni Novanta del secolo scorso [17, 18] e che hanno valutato le proporzioni esistenti fra diabete noto e diabete non diagnosticato permettono di stimare che almeno 1 milione di persone che vivono in Italia abbiano il diabete senza esserne a conoscenza. La somma dei casi noti e dei casi non diagnosticati porta il numero totale degli italiani con malattia a circa 5 milioni. Sarebbe auspicabile che l’identificazione dei casi di diabete non diagnosticato fosse un obiettivo per il nostro Sistema Sanitario Nazionale (SSN). Purtroppo non è così. Non esiste una progettualità di screening del diabete non diagnosticato, nonostante un costo decisamente contenuto.

**Figure 1:**
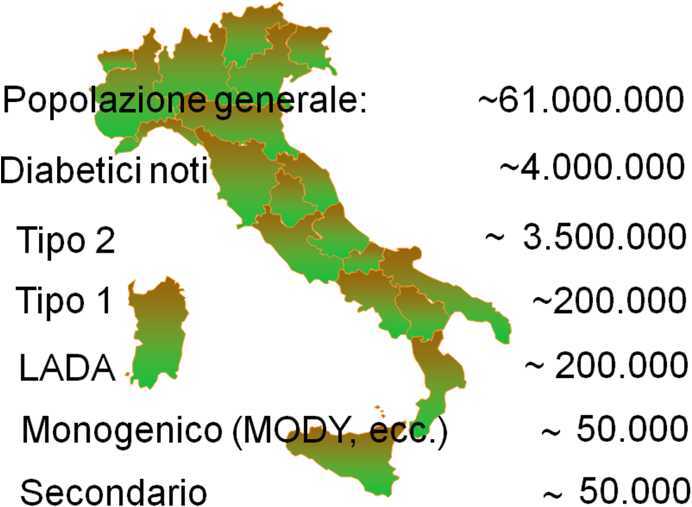

Dati amministrativi come quelli dell’Osservatorio ARNO non permettono di distinguere fra le varietà di diabete. Dati epidemiologici precedenti ci permettono di stimare che nel totale dei diabetici circa il 5% sia affetto da diabete tipo 1 [15, 16, 19]. La ricerca degli autoanticorpi specifici del diabete autoimmune fra i soggetti con diagnosi di diabete tipo 2 che vivono in Italia ha permesso di stabilire che la prevalenza del *Latent Autoimmune Diabetes of the Adult* (LADA) sia circa il 5% [20]. Altri studi condotti in Italia e in altre nazioni permettono di stabilire che i soggetti con diabete monogenico (es. MODY) sono poco più dell’1% dei casi e che quelli con diabete secondario (soprattutto pancreatogenico) abbiano una proporzione simile [21, 22]. La distribuzione dei casi di diabete nelle diverse varietà della malattia è riassunta nella Fig. 1: nella maggior parte dei casi si tratta di diabete tipo 2 ma il diabete autoimmune, nella variante classica (tipo 1) e meno classica (LADA), è presente in centinaia di migliaia di casi.

## Incidenza

**Figure 2:**
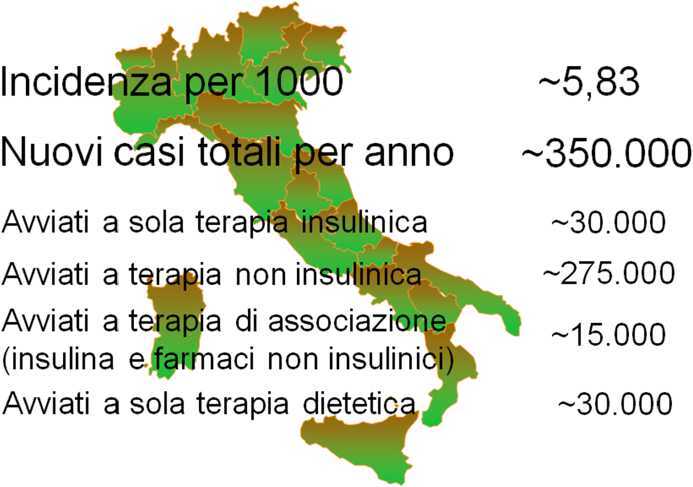

L’Osservatorio ARNO Diabete ha permesso anche di valutare l’incidenza della malattia [23]. Sono stati considerati casi incidenti i soggetti che per la prima volta nel 2018 hanno ricevuto la prescrizione di un farmaco per il diabete oppure l’esenzione per patologia o che hanno avuto una diagnosi di diabete sulla SDO senza che ci fossero precedenti noti di malattia. Incrociando le fonti sono stati individuati circa 66.000 casi, con un’incidenza di 5,83 per 1000 persone-anno (uomini 6,01 e donne 5,66 per 1000 persone-anno). Nella maggior parte dei casi la diagnosi coincideva con la prescrizione di un farmaco per il diabete che più spesso era un farmaco non insulinico (circa 85% dei casi). I dati amministrativi non consentono di distinguere fra i vari tipi di diabete ma è verosimile pensare che nella fascia di età 0–20 anni i casi avviati a terapia esclusivamente insulinica fossero quasi sempre bambini, ragazzi e adolescenti in cui era insorto diabete tipo 1. I casi incidenti avviati a terapia solo insulinica nelle altre fasce di età includevano certamente casi di diabete tipo 1, con un progressivo calo dei casi di questo tipo di diabete dopo i 40 anni, ma anche casi di diabete gestazionale (donne di età fra 20 e 40 anni) e diabete tipo 2 o di altro tipo (LADA, MODY, secondario, ecc.). I casi avviati a trattamento con farmaci non insulinici corrispondevano nella maggior parte dei casi a diabete tipo 2 ma fra essi, sicuramente, c’erano casi con altre varietà di diabete. È interessante rilevare la presenza di non pochi casi avviati a terapia non insulinica nella fascia di età 10–20 anni. Essi testimoniano un fenomeno osservato nelle ultime decadi, vale a dire l’identificazione di soggetti con un diabete diverso dal tipo 1 fra gli adolescenti. Si tratta con ogni probabilità di casi precoci di diabete tipo 2 oppure di diabete monogenico (es. MODY). L’estrapolazione dell’incidenza che abbiamo osservato all’intera popolazione italiana permette di stimare che i nuovi casi nell’anno siano circa 350.000, distribuiti nelle varie tipologia di terapia con le stime illustrate nella Fig. 2.

## Gravame della malattia

**Figure 3:**
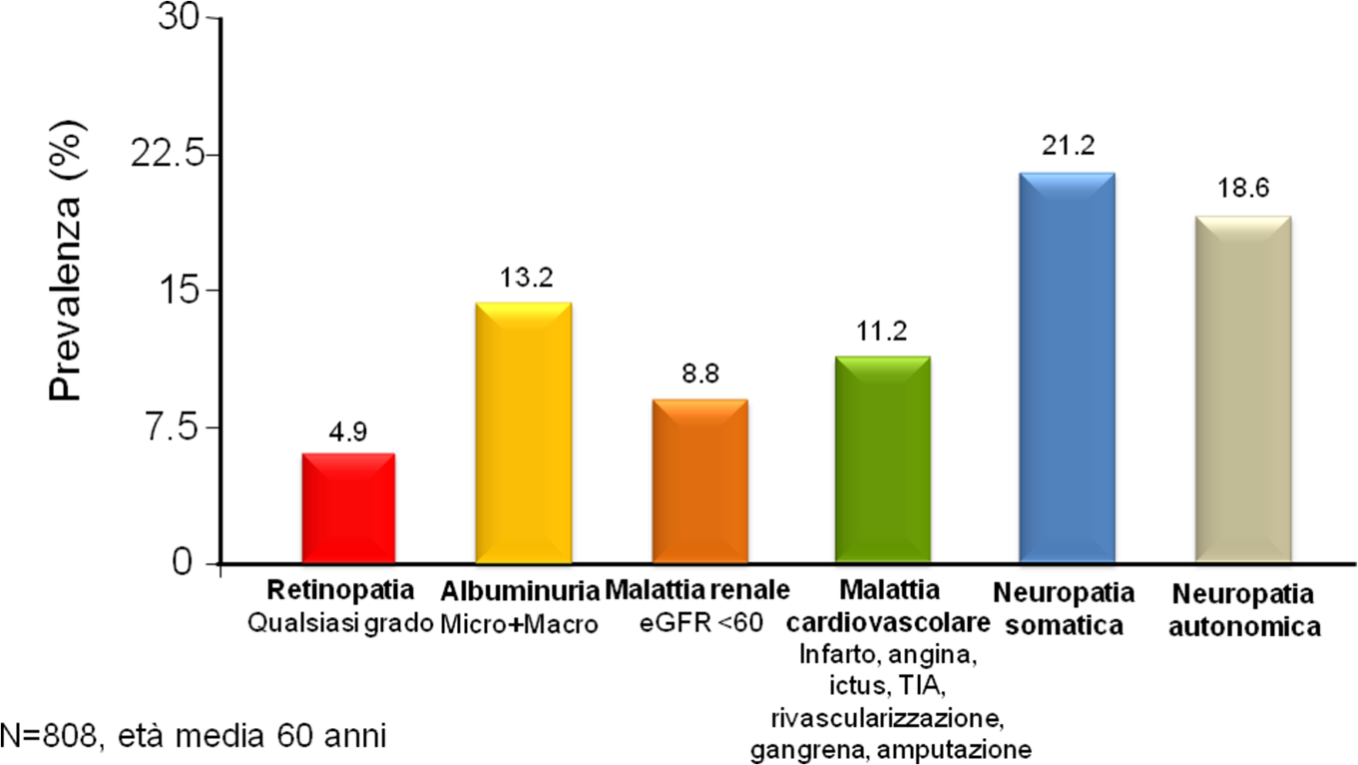

Alcuni studi epidemiologici condotti in Italia hanno documentato quanto siano prevalenti le complicanze croniche classiche del diabete (retinopatia, nefropatia, neuropatia, malattia cardiovascolare). Lo studio RIACE, sostenuto dalla Società Italiana di Diabetologia, ha esaminato i dati di circa 15 mila soggetti con diabete tipo 2 assistiti in una ventina di centri diabetologici universitari. L’età media era 67 anni e la diagnosi era avvenuta in media 11 anni prima (la durata della malattia nel diabete tipo 2 è difficile da definire). Lo studio delle complicanze ha documentato un’elevata e simile prevalenza (20–25%) di retinopatia, malattia renale cronica (eGFR <60), albuminuria (micro o macro), malattia cardiovascolare clinicamente manifesta (pregresso infarto, ictus, ecc.) [24, 25]. Questi dati, estrapolati alla popolazione con diabete che vive in Italia (circa 4 milioni di persone), permette di capire che le complicanze croniche interessano ben più di un milione di persone. È importante sottolineare che il danno d’organo a carico di occhio, rene, nervi, cuore e vasi nel diabete tipo 2 è già rilevabile al momento della diagnosi in moltissimi casi. In uno studio che abbiamo condotto a Verona su circa 800 soggetti con nuova diagnosi di diabete tipo 2 e in cui il danno d’organo è stato ricercato in maniera completa e approfondita, la prevalenza di retinopatia è risultata essere circa il 5% e quella di malattia renale, albuminuria e malattia cardiovascolare circa il 10% per ciascuna mentre quella di neuropatia somatica e autonomica era in entrambi i casi circa il 20% (Fig. 3) [26]. Nel complesso, al momento della diagnosi solo il 20% dei soggetti non risultava avere danno d’organo in alcuna sede, mentre l’80% di essi lo aveva in una o più sedi: microvascolare, macrovascolare o entrambi.

Uno studio epidemiologico condotto in Lombardia su dati amministrativi ha mostrato tassi di infarto e di ictus abbastanza simili in circa 180.000 persone con diabete di età compresa fra 45 e 84 anni (circa 9 e circa 8 eventi per 1000 persone-anno) con un rischio doppio rispetto alle persone senza il diabete [27]. Anche il rischio di mortalità per tutte le cause è risultato circa doppio nelle persone con diabete, con tassi di circa 34 decessi per 1000 persone-anno. Questo studio e altri condotti in Italia [28] hanno permesso di stimare che ogni anno muoiano in Italia circa 125.000 persone a causa o anche a causa del diabete. Le malattie cardiovascolari rappresentano circa il 35% di tutti i decessi [28].

**Figure 4:**
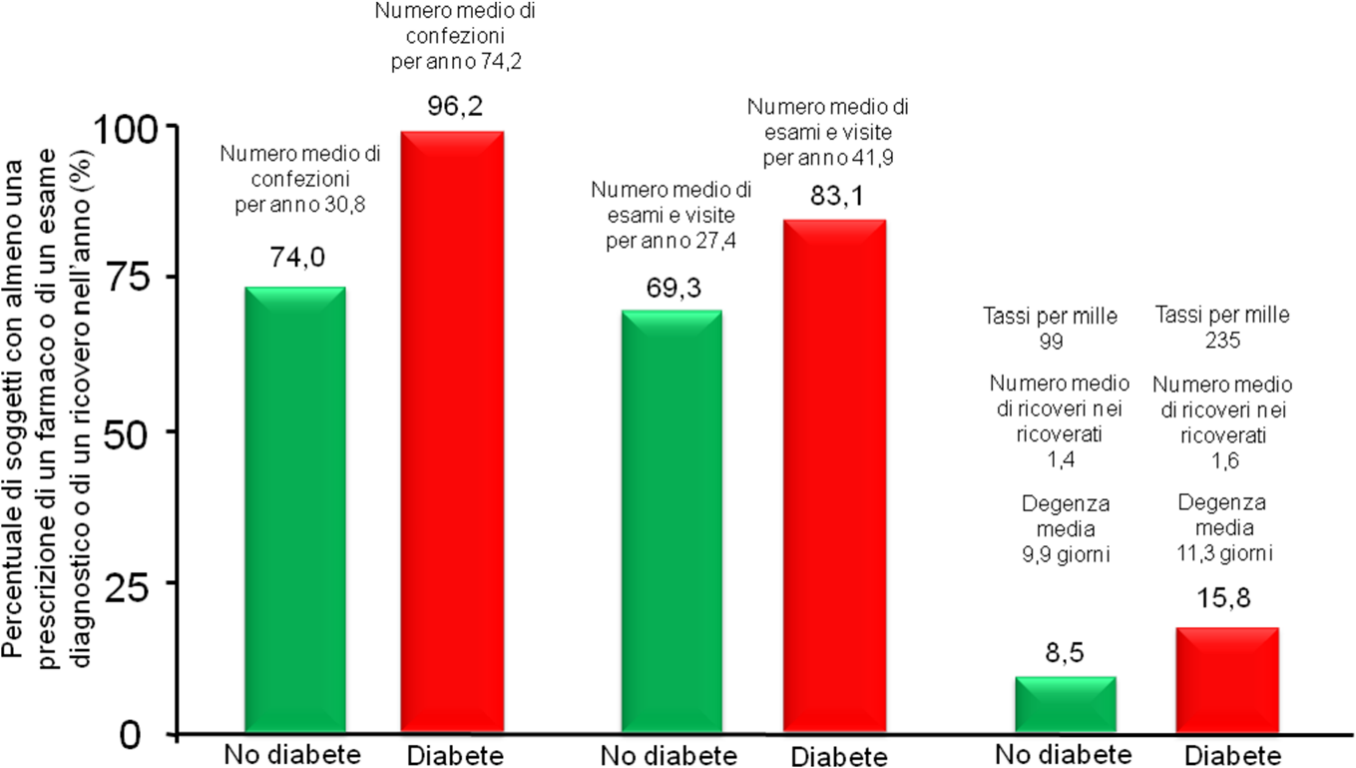

Il danno d’organo si traduce in un maggiore accesso a tutte le prestazioni sanitarie garantite dal nostro SSN [13]. Le persone con diabete nel 2018 hanno ricevuto la prescrizione con ricette SSN di un maggiore numero di farmaci rispetto alle persone senza il diabete, di esami di laboratorio, strumentali e di visite specialistiche. Praticamente tutti i farmaci sono stati prescritti nel 2018 in misura maggiore nelle persone con diabete, con l’unica eccezione dei farmaci per le malattie delle ossa. Anche tutti gli accertamenti diagnostici ambulatoriali sono stati prescritti in misura maggiore nelle persone con diabete, tranne la mammografia. Quest’ultimo dato è preoccupante, considerando che praticamente tutti i tumori, comprese le neoplasie mammarie, hanno un’incidenza più alta nelle persone con diabete [29]. Anche i ricoveri ospedalieri sono stati più frequenti nelle persone con diabete, interessandone il 16% circa contro il 9% circa nelle persone senza il diabete e con tassi di 235 rispetto a 99 per 1000 persone-anno (Fig. 4).

Tutte le patologie sono state causa di ricovero in misura maggiore nelle persone con diabete. Fra le cause più frequenti di ospedalizzazione spiccano lo scompenso cardiaco, la patologia ischemica coronarica o cerebrale, le aritmie, l’insufficienza renale acuta e cronica. Nel complesso, le malattie cardiovascolari hanno costituito circa il 20% delle cause di ricovero. Estrapolando i dati su base nazionale, si può stimare che ogni anno in Italia ci siano fra i diabetici circa 80.000 ricoveri per scompenso cardiaco, 40.000 per infarto del miocardio, 30.000 per ictus, 10.000 per amputazione (di cui 3.000 maggiori). In Italia si registrano anche ogni anno circa 2.000 casi incidenti di terapia dialitica.

I costi della malattia ammontano a circa 4.000 euro per paziente, di cui solo il 10% per la gestione standard (visite specialistiche, esami di laboratorio per il monitoraggio metabolico, farmaci anti-iperglicemici, dispositivi per monitoraggio e terapia come strisce reattive, lancette e aghi) e circa il 90% per le complicanze (ricoveri ospedalieri, diagnostica ambulatoriale, farmaci). In totale, si tratta di circa 16 miliardi di euro per anno, a cui aggiungere una pari somma costituita dai costi diretti personali (es. pagamento di ticket o di visite extra SSN) e dai costi indiretti (es. pensione anticipata, invalidità, assenze dal lavoro, ecc.) [30].

## Qualità della cura

La valutazione di indicatori riguardanti la qualità della cura mostrano che questa è subottimale, soprattutto nelle persone che non ricevono assistenza specialistica [31]. Questa è stata identificata dal riscontro di almeno una ricetta per visita endocrinologica o diabetologica nell’anno. Le persone con diabete che non hanno avuto assistenza specialistica sono circa il 70% (la stima è di circa 2,8 milioni). Da notare che queste persone non risultano essere diverse da chi ha ricevuto assistenza specialistica (circa il 30%, pari a 1,2 milioni) quando vengono confrontate per la presenza di elevato rischio cardiovascolare (esenzioni per patologia, ricoveri per malattie cardiovascolari, uso di anti-aggreganti piastrinici). Il confronto fra chi ha ricevuto e chi non ha ricevuto assistenza specialistica nell’anno 2018 mostra chiaramente che la qualità della cura, già subottimale in chi ha ricevuto assistenza specialistica, è stata assai precaria in chi non l’ha ricevuta. Come mostrato nelle Figg. 5 e 6, sia i parametri di laboratorio essenziali (es. HbA1c, colesterolo, creatinina), sia gli esami strumentali raccomandati (es. ECG, ecodoppler carotideo o degli arti inferiori) sono stati prescritti con frequenza decisamente inferiore al raccomandato ai pazienti che non hanno ricevuto assistenza specialistica [31]. Questi ultimi hanno anche ricevuto minori prescrizioni di farmaci anti-ipertensivi, ipolipidemizzanti e anti-aggreganti piastrinici. Inoltre, per questioni legate alle autorizzazioni dell’Agenzia Italiana del Farmaco (AIFA), i soggetti che non hanno ricevuto assistenza specialistica non hanno potuto accedere alle prescrizioni dei farmaci più moderni per la cura del diabete anche quando i medesimi erano fortemente raccomandati per esercitare un’efficace cardio-protezione e nefro-protezione [31]. Da notare che la prescrizione dei cosiddetti “nuovi farmaci per il diabete” (DPP-4 inibitori, SGLT-2 inibitori, analoghi GLP-1) ha interessato quasi il 100% dei soggetti che hanno ricevuto assistenza specialistica. Tuttavia, anche nelle strutture specialistiche la prescrizione di farmaci cardioprotettori (SGLT-2 inibitori e analoghi GLP-1) nei soggetti con malattia cardiovascolare nota o con alto rischio è stata inferiore all’atteso e non ha raggiunto nel suo complesso il 50% [31].

**Figure 5:**
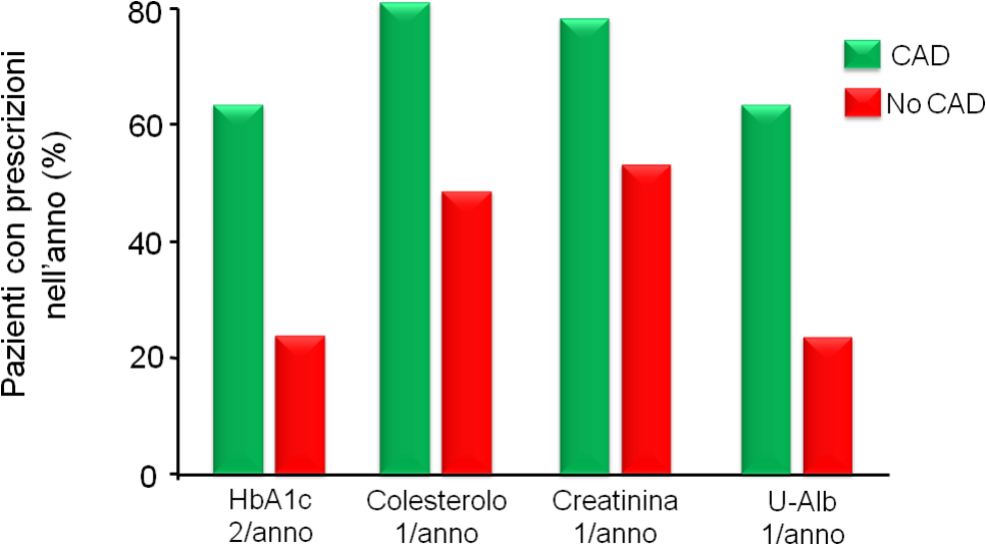

**Figure 6:**
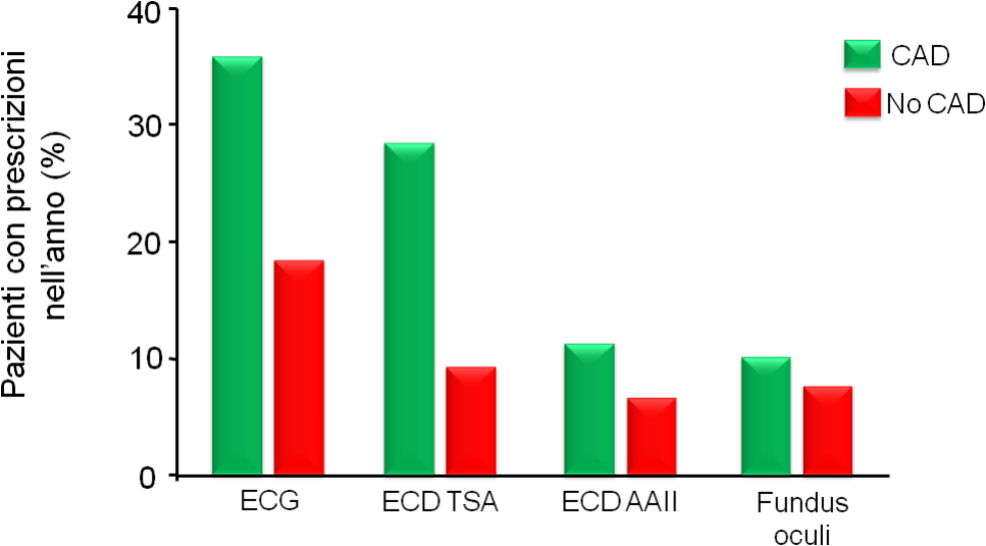

## Organizzazione dell’assistenza diabetologica in Italia fra virtù e trascuratezze

**Figure 7:**
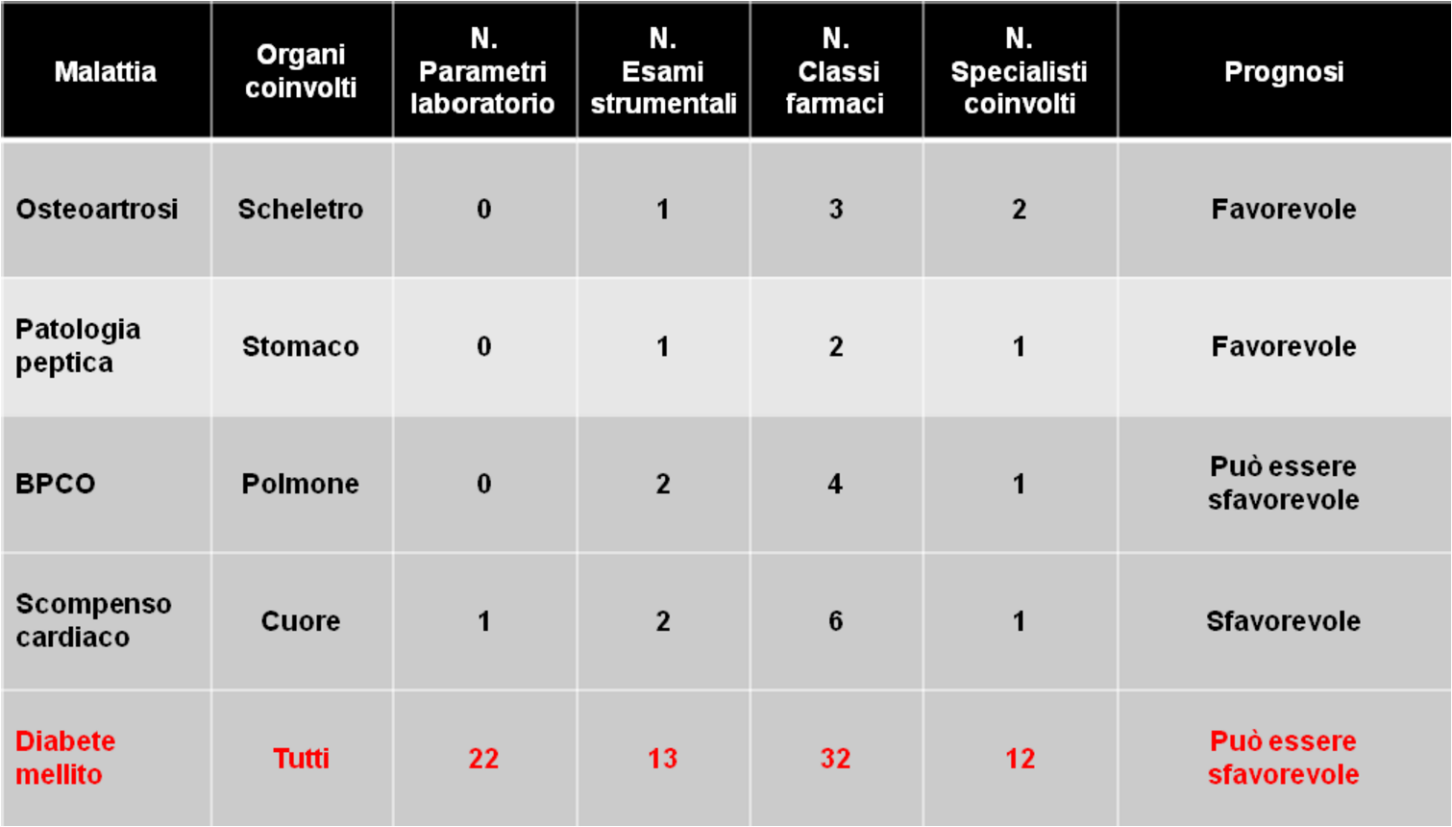

L’Italia è il primo Paese al mondo in cui è stata promulgata una legge specifica riguardante il diabete. Era il 16 marzo 1987 quando fu approvata dal Parlamento la legge n. 115 che sanciva, fra l’altro, il diritto delle persone con diabete a ricevere assistenza da parte di una rete di strutture specialistiche. Il Piano sulla Malattia Diabetica approvato dalla Conferenza Stato-Regioni il 6 dicembre 2012 ha confermato questo principio e ha incluso il principio che in Italia ogni persona con diabete debba essere assistita anche presso le strutture diabetologiche fin dal momento della diagnosi, con una presa in carico condivisa da parte del medico di medicina generale e dal team diabetologico. Si tratta di quella gestione integrata della patologia diabetica di cui si parla da anni e che talora viene propagandata come realmente esistente in qualche ambito territoriale anche se questo non corrisponde affatto alla verità. Una gestione integrata che, propugnata da tutti (specialisti e medici di famiglia, amministratori della sanità e politici), dovrebbe prevedere dialoghi frequenti fra i vari professionisti coinvolti e interventi specialistici calibrati nel tempo in funzione delle necessità dettate dalla tipologia della malattia e dalla sua evoluzione nel tempo. Il diabete, diversamente da altre patologie croniche, è una malattia sistemica e non mono-organo o mono-apparato, notevolmente eterogenea nella sua espressione clinica, estremamente complessa nella sua gestione che prevede molti esami di laboratorio e strumentali fra cui destreggiarsi, molte classi di farmaci fra cui scegliere, molti professionisti con cui interagire (Fig. 7).

Per questo motivo, il ruolo del team diabetologico dovrebbe essere ritenuto imprescindibile anche oggi, a distanza di 35 anni dalla legge 115 del 1987. Tale imprescindibilità in effetti appare in tutti documenti che hanno avuto come oggetto la malattia redatti a livello nazionale, regionale o locale. Ci sono regioni italiane che hanno legiferato in tal senso o che hanno prodotto inequivocabili raccomandazioni, documenti di indirizzo, PDTA. Nonostante tutto questo, ancora manca la piena applicazione di quanto previsto dalle leggi e da questi documenti. Di conseguenza, esiste una chiara e inaccettabile disuguaglianza fra i cittadini che sono assistiti anche dalle strutture diabetologiche (circa 1 su 3) e quelli che non lo sono (circa 2 su 3). Una disuguaglianza che causa non solo quelle diversità nella qualità della cura di cui si è scritto in precedenza ma anche un dato drammatico che riguarda un indicatore che non lascia adito a interpretazioni: la mortalità.

Almeno tre studi italiani e una recente meta-analisi dei medesimi hanno documentato che l’assistenza diabetologica si traduce in una riduzione della mortalità per tutte le cause del 19% [32]. Il calcolo del NNT per la prevenzione di un decesso con una visita diabetologica per anno mostra un risultato particolarmente favorevole (circa 150 soggetti da trattare). Da notare che il NNT è simile a quello calcolato per il trattamento con farmaci considerati salvavita come le statine o gli ACE inibitori in prevenzione secondaria, con un costo minore (circa 40 euro). Non è improprio affermare che la visita diabetologica è una “procedura salvavita”.

Dagli anni Settanta del secolo scorso l’Italia si è dotata di una rete sempre più capillare di strutture diabetologiche. Una recente indagine della Società Italiana di Diabetologia ne ha identificate circa 650, con una distribuzione piuttosto disomogenea fra le regioni ed entro le regioni. La metà di esse, tuttavia, non consiste in centri propriamente detti, dotati di risorse multiprofessionali (medici specialisti, infermieri, dietisti, eventualmente psicologi e podologi, collaborazioni strette con altri specialisti come oculisti, nefrologi, cardiologi, ecc.) ma piuttosto in ambulatori dove operano specialisti isolati. Questa tipologia di assistenza specialistica non rappresenta quella ideale, così come sottolineato anche nel Piano della Malattia Diabetica. Solo il team diabetologico è, infatti, in grado di offrire quegli interventi multiprofessionali e multidimensionali che sono ritenuti indispensabili per garantire esiti migliori. Lo specialista isolato non riesce a esprimere tutte le competenze necessarie per affrontare compiutamente la complessità del diabete. L’idea di chi scrive e di molti colleghi che operano da anni nel contesto diabetologico italiano è che, approfittando anche delle opportunità offerte dal PNRR, debba essere operato un intervento di razionalizzazione delle strutture di diabetologia italiane, con accorpamento dei singoli specialisti presso i centri diabetologici propriamente detti, dotando contemporaneamente questi ultimi di tutte le figure professionali necessarie.

Al momento attuale nel sistema SSN operano circa 2000 specialisti in diabetologia, con un rapporto di 1 ogni 2000 persone con diabete. Non pochi di questi specialisti operano solo parzialmente nell’assistenza alle persone con diabete, in quanto impegnati anche in altre attività ambulatoriali o di degenza. Molti di loro, fra l’altro, sono impegnati nella cura della podopatia diabetica e della gravidanza nel diabete, nell’uso della tecnologia per la cura del diabete tipo 1, nello screening/stadiazione delle complicanze croniche della malattia, dedicando sempre meno tempo alla gestione standard della malattia. La disponibilità di infermieri e dietisti è ancora più critica con carenze diffuse in termini numerici e di attività a tempo pieno nel contesto diabetologico. Si calcola che gli infermieri esperti di diabete non siano più di 2000 e i dietisti con competenze diabetologiche non più di 500. Gli psicologi e i podologi nelle strutture diabetologiche sono un’assoluta rarità, contandosi in poche decine. Ne consegue che l’intervento riorganizzativo dell’assistenza diabetologica in Italia dovrebbe essere profondo: ridurre il numero dei centri è razionale e auspicato, prevedendone 1 ogni 15 mila pazienti, ma specialisti, infermieri e dietisti operanti nella rete dei centri dovrebbero raddoppiare (da 2000 a 4000 diabetologi, da 2000 a 4000 infermieri, da 500 a 1000 dietisti) e in ogni centro dovrebbe idealmente essere inserito un podologo e uno psicologo. I centri potrebbero in tal modo garantire sia un dialogo stretto di tipo digitale (teleconsulto) con la medicina generale e anche una presenza di proprio personale nelle Case della Salute (o della Comunità) quando necessario. La rete diabetologica italiana andrebbe potenziata e non smantellata, come avvenuto in alcune realtà.

## Conclusioni

La pandemia diabete si esprime anche in Italia con numeri impressionanti. L’assistenza alle persone con diabete in Italia è fra le migliori del mondo, come testimoniato da numerosi indicatori (livello medio di HbA1c, tassi di ricovero per scompenso metabolico, epidemiologia delle complicanze acute, mortalità), ma esiste un margine rilevante di miglioramento. È soprattutto necessario eliminare le disuguaglianze e le iniquità esistenti fra chi riceve assistenza specialistica e chi non la riceve mai e fra chi riceve assistenza da parte di centri specialistici con multiple competenze e chi la riceve invece da parte di professionisti isolati. Le persone con diabete hanno un bisogno insopprimibile di aumentare la qualità e la quantità della loro vita e questo può essere ottenuto grazie a una rete di centri specialistici sempre più incisivi ed efficaci nella loro azione. Il diabete si cura con esperienza, competenza e dedizione e lo squilibrio attualmente esistente fra numero di pazienti e numero di professionisti esperti, competenti e dedicati va corretto.

## Informazioni Supplementari

I link al materiale elettronico supplementare sono elencati qui sotto.

## Dichiarazioni etiche

### Conflitto di interesse

L’autore Enzo Bonora dichiara di non avere conflitti di interesse.

### Consenso informato

Lo studio presentato in questo articolo non ha richiesto sperimentazione umana

### Studi sugli animali

L’autore di questo articolo non ha eseguito studi sugli animali.
