# Supplementary material for: La pandemia diabete in Italia
Source: L'Endocrinologo. 2022 Jul 27;23(4):337–44. [Article in Italian] doi: 10.1007/s40619-022-01130-4 (PMC9327878; doi:10.1007/s40619-022-01130-4)
Supplement: Supplementary file 1 [file 40619_2022_1130_MOESM1_ESM.doc]

**Scheda di autovalutazione**

**1. La prevalenza del diabete noto in Italia è circa:**

a. 4%

b. 5%

c. 6%

d. 7%

**2. L’incidenza del diabete noto in Italia è circa:**

a. 150 per 100 mila

b. 250 per 100 mila

c. 350 per 100 mila

d. 450 per 100 mila

**3. Al momento della diagnosi i soggetti con diabete tipo 2 che non hanno danno d’organo a carico di cuore, vasi, reni, occhi o nervi sono circa:**

a. 20%

b. 30%

c. 40%

d. 50%

**4. In Italia le persone con diabete che sono assistite nei centri diabetologici sono attualmente circa:**

a. 30%

b. 60%

c. 50%

d. 40%
