# Supplementary material for: La pandemia diabete in Italia
Source: L'Endocrinologo. 2022 Jul 27;23(4):337–44. [Article in Italian] doi: 10.1007/s40619-022-01130-4 (PMC9327878; doi:10.1007/s40619-022-01130-4)
Supplement: Supplementary file 2 [file 40619_2022_1130_MOESM2_ESM.doc]

**RISPOSTE**

**1. La prevalenza del diabete noto in Italia è circa:**

c. 6%

**2. L’incidenza del diabete noto in Italia è circa:**

c. 350 per 100 mila

**3. Al momento della diagnosi i soggetti con diabete tipo 2 che non hanno danno d’organo a carico di cuore, vasi, reni, occhi o nervi sono circa:**

a. 20%

**4. In Italia le persone con diabete che sono assistite nei centri diabetologici sono attualmente circa:**

a. 30%
